# Ontogeny of Endocannabinoid Modulation of Neuromuscular Transmission: Contribution of Postsynaptic Nicotinic Receptors and Butyrylcholinesterase-Sensitive Mechanisms

**DOI:** 10.3390/cells15171524

**Published:** 2026-08-24

**Authors:** Egor Nevsky, Oksana Lenina, Irina Zueva, Dmitry Samigullin, Artem Malomouzh, Vladimir Parpura, Konstantin Petrov

**Affiliations:** 1 Kazan Institute of Biochemistry and Biophysics, FRC Kazan Scientific Center of RAS, 420111 Kazan, Russia; nevskywissen@gmail.com (E.N.); samid75@mail.ru (D.S.); artur57@list.ru (A.M.); 2 Institute of Fundamental Medicine and Biology, Kazan Federal University, 420008 Kazan, Russia; 3 Arbuzov Institute of Organic and Physical Chemistry, FRC Kazan Scientific Center of RAS, 420088 Kazan, Russia; leninaox@mail.ru (O.L.); zueva.irina.vladimirovna@gmail.com (I.Z.); 4 Department of Radiophotonics and Microwave Technologies, Kazan National Research Technical University Named After A.N. Tupolev-KAI, 420111 Kazan, Russia; 5 International Translational Neuroscience Research Institute, Zhejiang Chinese Medical University, Hangzhou 310053, China

**Keywords:** neuromuscular junction, acetylcholine release, butyrylcholinesterase, cannabinoid receptors, newborn mice, synaptic homeostasis

## Abstract

**Highlights:**

**What are the main findings?**

- CB1 receptors modulate acetylcholine release only in developing synapses and require postsynaptic nicotinic receptors.
- Butyrylcholinesterase degrades endocannabinoids to tune this signaling.

**What are the implications of the main findings?**

- This presynaptic–postsynaptic coupling reveals a novel retrograde homeostatic mechanism essential for synapse maturation.
- Non-canonical endocannabinoid degradation by butyrylcholinesterase highlights age-specific regulators of motor control.

**Abstract:**

Endocannabinoid receptors of the CB1 subtype are the most abundant G-protein-coupled receptors in the central nervous system, where they strongly regulate neurotransmitter release. The effects of the activation of these receptors by exogenously applied agonists have also been described in the peripheral nervous system, particularly at neuromuscular junctions (NMJs). However, the physiological role of these receptors at NMJs has not been demonstrated. We have shown that blockade of CB1 receptors at the NMJs of newborn or young mice increases the quantal content of end-plate potentials, as well as their decay time constant. Neither effect of CB1 receptor blockade is observed if postsynaptic muscle acetylcholine receptors are partially blocked. Thus, CB1 receptors may be involved in maintaining synaptic homeostasis. Importantly, the effect of CB1 receptor blockade on quantal content is potentiated by blockade of the enzyme butyrylcholinesterase. Therefore, butyrylcholinesterase may be considered a component of the extracellular degradation system for endocannabinoids.

## 1. Introduction

The endocannabinoid (eCB) system comprises endogenous lipid mediators, their receptors, and the enzymatic machinery responsible for their synthesis and degradation [1]. Among the best-characterized eCBs are anandamide (AEA) and 2-arachidonoylglycerol (2-AG), which are synthesized on demand from membrane phospholipid precursors and primarily act through cannabinoid receptors. The canonical cannabinoid receptors CB1 and CB2 are G_i/o_ protein-coupled receptors that exhibit constitutive activity and regulate multiple intracellular signaling pathways [2]. A growing body of research has demonstrated that the endocannabinoid (eCB) signaling system is critically involved in multiple stages of organismal development, spanning from early embryonic patterning and the assembly of neural networks to the establishment of synaptic connections and the postnatal refinement of nervous system architecture [1,2,3].

In the nervous system, endocannabinoids function predominantly as retrograde messengers that modulate neurotransmitter release and synaptic plasticity [4]. While the mechanisms of eCB signaling have been extensively investigated in central synapses, considerably less is known about their role at peripheral cholinergic synapses, including the neuromuscular junction (NMJ). Early studies demonstrated that exogenous cannabinoids can alter neurotransmission at amphibian NMJs. For example, AEA abolished cAMP-dependent facilitation of transmitter release at the frog NMJ [5], whereas CB1 receptor agonists were later shown to exert bidirectional effects on evoked acetylcholine (ACh) release [6]. More recently, exogenous application of AEA and 2-AG was shown to potentiate ACh release at mouse motor synapses through noncanonical mechanisms, indicating that cannabinoid signaling can modulate neurotransmission at mammalian NMJs [7].

Evidence for eCB signaling at the NMJ is considerably more limited. Muscarinic receptor activation induces endocannabinoid-dependent depression of neurotransmitter release through presynaptic CB1 receptors at the lizard NMJ, providing the first direct evidence for physiological eCB regulation of neuromuscular synaptic transmission [8]. However, the conditions under which the eCB system can regulate transmitter release at mammalian NMJs remain unknown.

The postnatal period is characterized by profound structural and functional remodeling of mammalian NMJs, including the maturation of neurotransmitter release mechanisms, elimination of redundant synaptic inputs, and replacement of fetal (γ-containing) nicotinic ACh receptors (nAChRs) with the adult ε-containing receptor isoform [9,10,11]. Given the established role of the eCB system in neural development, it is plausible that eCB signaling contributes to the maturation and functional regulation of developing NMJs [1,2,3,12]. Therefore, the purpose of this study was to test the activity of the eCB system at the NMJs of newborn and young mice.

To investigate the contribution of the eCB system and cholinergic mechanisms to NMJ function, we used four pharmacological agents with well-characterized targets. AM-251 is a potent CB1 receptor antagonist/inverse agonist [13] with high affinity for CB1 receptors (Ki = 7.49 nM) and substantially lower affinity for CB2 receptors (Ki = 2290 nM) [14]. However, AM-251 can have off-target effects at higher concentrations, including positive allosteric modulation of GABA_A_ receptors, which should be considered when pharmacological experiments are being performed [15]; consequently, we did not utilize AM-251 at concentrations high enough to have off-target effects. Bambuterol is a carbamate prodrug that acts as a potent and relatively selective inhibitor of butyrylcholinesterase (BChE), with considerably lower potency toward acetylcholinesterase [16]. d-Tubocurarine (dTc) is a classical competitive antagonist of nAChRs [17,18]. Finally, Waglerin-1 is a peptide toxin that selectively inhibits the adult ε-containing form of the muscle nAChR. Its selectivity is developmentally relevant because neonatal NMJs predominantly express γ-containing nAChRs, whereas the ε-containing receptor becomes predominant during postnatal maturation [19]. Thus, the combined use of AM-251, bambuterol, dTc, and Waglerin-1 allowed us to pharmacologically distinguish the contributions of CB1 signaling, BChE activity, overall nAChR-mediated transmission, and specifically the adult ε-containing nAChR isoform to NMJ function during postnatal development.

CB1 receptor blockade has been shown to increase the level of ACh release at mouse NMJs on the second and fourteenth days of postnatal development. In addition, CB1 receptor blockade increases the decay time constant of end-plate potentials (EPPs). Interestingly, the effects of eCB receptor blockade depend on the activity of adult (ε-containing) nAChRs and BChE activity. We also showed that CB1 receptor blockade increases the rate of muscle fatigue ex vivo.

## 2. Materials and Methods

### 2.1. Ethical Approval and Animals

All procedures involving animals were conducted in strict compliance with the ARRIVE guidelines and NIH Guide for the Care and Use of Laboratory Animals as well as the requirements set forth in EU Directive 2010/63/EU. The experimental protocols received approval from the Animal Care and Use Committee affiliated with the FRC Kazan Scientific Center of the Russian Academy of Sciences (protocol No. 23/7, dated 12 May 2023). Every reasonable measure was taken to reduce distress and discomfort experienced by the animals. CD-1 mice were housed in plastic cages containing sawdust bedding and maintained in a room with adequate ventilation at a temperature of 20–22 °C, a 12 h light/dark cycle, and relative humidity of 60–70%. Food and water were provided without restriction.

Experiments were conducted on isolated neuromuscular preparations obtained from the m. levator auris longus (LAL) [13] and the diaphragm. Tissues were harvested from the animals at postnatal day 2 (P2), postnatal day 14 (P14), and two months of age (P60). Prior to tissue extraction, each animal was subjected to deep anesthesia using isoflurane, followed by rapid decapitation. No deliberate randomization of animals within each age group was performed, and the researchers were not blinded with respect to the identity of the applied substances.

### 2.2. Chemicals

The CB1 receptor blockers AM-251 (Cat. # A6226), bambuterol (Cat. # B8684), and d-tuborurarine (Cat. #5.05145) were purchased from Sigma–Aldrich (St. Louis, MO, USA). Waglerin-1 (Cat. # STW-001) was purchased from Alamone Labs (Jerusalem, Israel).

### 2.3. Electrophysiological Recordings

The experimental procedure followed the protocol outlined in our earlier publication [20]. Isolated LAL nerve–muscle preparations were secured with pins in transparent recording chambers and continuously superfused (2–3 mL min^−1^) with Krebs–Ringer solution equilibrated with a gas mixture of 95% O_2_ and 5% CO_2_. The physiological solution had the following composition (mM): NaCl—137, KCl—5, CaCl_2_—2, MgCl_2_—1, Na_2_HPO_4_—1, NaHCO_3_—11.9, and glucose—11, adjusted to pH 7.4.

Stock solutions of the compounds under investigation were prepared in distilled water at concentrations ranging from 1 to 10 mM, with the exception of AM-251, which was dissolved in DMSO to yield a 10 mM stock. The pH of all aqueous stock solutions was maintained within the range of 7.2–7.4. The prepared stocks were stored at −20 °C until needed, while the AM-251 stock was kept at room temperature. Immediately prior to each experiment, the appropriate stock was diluted in Krebs–Ringer solution to reach final working concentrations of 50 nM, 100 nM, 1 μM, or 5 μM. The DMSO content in the bathing solution never exceeded 0.5% (*v*/*v*).

End-plate potentials (EPPs) and spontaneous miniature EPPs (mEPPs) were registered at 20–22 °C with conventional glass microelectrodes (tip resistance 10–15 MΩ) back-filled with 3 M KCl. Because µ-conotoxin GIIIB lacks efficacy in neonatal preparations [21], muscle fiber contraction and action potential generation during EPP/mEPP recordings were prevented by making transverse cuts through the muscle fibers [22]. The motor nerve was stimulated through a suction electrode delivering supramaximal current pulses of 0.1 ms duration at a repetition rate of 0.5 Hz. Recordings were obtained from 5 to 10 end-plate regions prior to drug application and from a separate set of 5–10 end-plates following treatment. At each end-plate, 50 EPPs and 50 mEPPs were captured. Quantal content (QC) was estimated as the ratio of the average EPP amplitude to the average mEPP amplitude measured at the same junction. Only muscle fibers exhibiting a stable resting membrane potential of −40 ± 2 mV were included in the analysis. To account for the nonlinear summation of EPP and mEPP amplitudes, Martin’s correction factor was applied [23].

Synaptic signals were acquired with an Axoclamp 900A amplifier, digitized via a Digidata 1440A interface (Molecular Devices, San Jose, CA, USA), and subsequently processed using pClamp v. 10.4 software (Molecular Devices, USA).

### 2.4. Recordings of Muscle Contractile Responses

Hemi-diaphragm preparations together with their intact phrenic nerves were immersed in continuously oxygenated Krebs–Ringer solution maintained at 25 °C. Isometric twitch force was monitored using a TRI201AD force transducer (AD Instruments, Sydney, NSW, Australia). Muscle contractions were elicited by applying supramaximal current pulses (0.1 ms pulse width) to the phrenic nerve through a suction electrode. Stimulation trains were delivered at frequencies of 1, 10, 20, 40, 70, and 100 Hz, each lasting 30 s, with a 2 Min rest period separating consecutive trains. The optimal resting length of the muscle and the appropriate stimulation voltage were established by incrementally adjusting the muscle length with a micromanipulator until maximal force output was achieved. All the force recordings were acquired and stored using a PowerLab data-acquisition system running LabChart 6 software (ADInstruments, Colorado Springs, CO, USA).

### 2.5. Histochemical Detection of Butyrylcholinesterase Activity

After dissection, the LAL muscle samples were fixed in 4% paraformaldehyde for 24 h at 4 °C and then transferred to 30% sucrose in phosphate-buffered saline (PBS, pH 7.4) containing 0.02% sodium azide. Histochemical staining for BChE was performed as described previously [24] with modifications. Briefly, the samples were washed in 0.1 M phosphate buffer (pH 7.4) for 30 min and incubated for 30 min in 0.1 M phosphate buffer (pH 7.4) supplemented with 0.3% H_2_O_2_. A subset of samples was incubated for 30 min in 0.1 M maleate buffer (pH 7.0) supplemented with 100 nM bambuterol as a BChE inhibitor, while the remaining samples were incubated in 0.1 M maleate buffer (pH 7.0) without bambuterol. The samples were subsequently transferred for 1.5 h to a reaction mixture containing 0.1 M maleate buffer (pH 7.0), 5 nM C547 as a specific acetylcholinesterase inhibitor [25], 0.05 mM K_3_Fe(CN)_6_, 0.47 mM CuSO_4_, 0.5 mM ammonium citrate, and 0.8 mM butyrylthiocholine as a substrate for BChE. In the experimental group, 100 nM bambuterol was added to the mixture, whereas the control sections were held in maleate buffer. After the enzymatic reaction, the samples were washed in distilled water for 30 min, incubated for 10 min in 0.3% nickel(II) ammonium sulfate solution, washed in distilled water for 30 min, and placed in 1.39 mM 3,3′-diaminobenzidine tetrahydrochloride in 0.1 M phosphate buffer (pH 7.4) for 5 min, after which the staining was developed by adding 0.3% H_2_O_2_ for 3 min. The reaction was stopped by sequential washing first in 0.01 M acetate buffer (pH 3.3) and then in distilled water. Micrographs of the preparations were acquired using an Olympus IX83 inverted microscope (Evident, Tokyo, Japan). A total of five control muscles and five bambuterol-treated muscles were used for histochemical detection of butyrylcholinesterase activity in each age group.

### 2.6. Statistics

The number of samples necessary for each experimental series was determined in advance through power analysis, with the statistical power set at 80% and a significance threshold of α = 0.05. All the statistical procedures were carried out in OriginPro 2021b (OriginLab, Northampton, MA, USA). The results are expressed as the mean values ± standard errors of the mean (SEM). The sample size (n) corresponds to the total number of NMJs recorded, derived from N individual animals, as specified in the respective figure legends. No data points were removed as outliers prior to analysis.

The distribution of the data was evaluated for normality by means of the Shapiro–Wilk test. Differences between two experimental groups were assessed using one-way ANOVA. Changes in muscle contraction force were evaluated with a paired Student’s *t*-test. A *p*-value less than 0.05 was accepted as the criterion for statistical significance. Significance levels are denoted throughout the figures as follows: * *p* < 0.05, ** *p* < 0.01, and *** *p* < 0.001.

## 3. Results

### 3.1. CB1 Receptors Mediate the Modulation of EPP Quantal Content and Decay Time Constant in Newborn, Young but Not in Adult Mice

To evaluate the potential synaptic effects of a CB1 blocker (AM-251, 5 μM), we analyzed the QC, amplitude, rise time and decay time constant of EPPs in newborn, young and adult mice.

**Figure 1:**
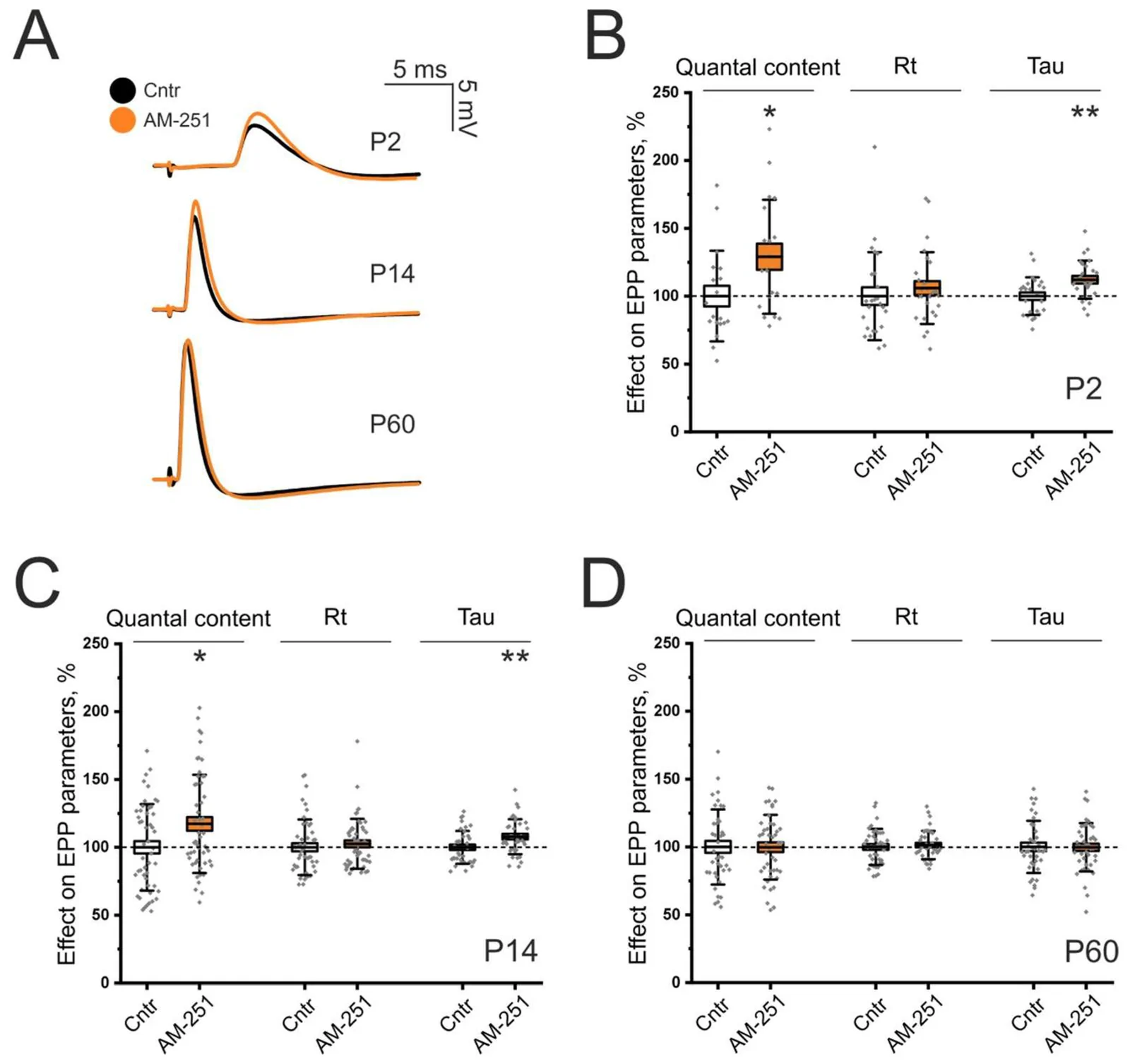
Effects of the CB1 antagonist AM-251 on evoked acetylcholine release at LAL NMJ. (**A**) Representative EPP recordings at NMJs in the control group (Cntr) and following application of the CB1 antagonist AM-251. (**B**) Effect of AM-251 (5 μM) on EPP parameters (quantal content (QC), rise time (RT) and decay time constant (Tau)) at P2. (**C**) Effect of AM-251 on EPP parameters at P14. (**D**) A lack of AM-251 effect on EPP parameters at P60. *n* = 20–43 NMJs; N = 5–7 animals; * *p* < 0.05; ** *p* < 0.01 vs. control values (one-way ANOVA).

The average QC and decay time constant in newborn (P2) animals were 2.59 ± 0.2 and 7.29 ± 0.2 ms, respectively, and were taken as 100%. In this age group, we observed the strongest effect of AM-251, which increased the QC and decay time constant by 29 ± 9.63% (*p* < 0.05; Figure 1A,B) and 12.12 ± 2.71% (*p* < 0.01; Figure 1A,B), respectively.

In turn, in a young synapse (P14), the average QC, amplitude of EPPs and decay time constant were 5.5 ± 0.25, 8.48 ± 0.63 mV and 2.6 ± 0.05 ms, respectively. The application of AM-251 significantly increased the QC and decay time constant by 17.24 ± 4.9% (*p* < 0.05; Figure 1A,C) and 7.84 ± 2.05% (*p* < 0.01; Figure 1A,C), respectively.

The average amplitude of EPPs in the control at P60 was 16.2 ± 1 mV, and that in the QC was 19.7 ± 0.9. The application of AM-251 (5 μM) did not alter the QC (99.8 ± 3.6%, *p* = 0.93; Figure 1A,D) or the amplitude, rise time, or decay time constant of the EPPs (Figure 1D).

Thus, blockage of CB1 receptors increases evoked ACh release at both P2 and P14 NMJs. In addition, CB1 receptor inactivation increases the decay time constant of EPP at these developmental stages. Notably, the effects of eCB receptor blockers decreased from P2 to P14.

### 3.2. Effects of CB1 Receptor Activation Depend on the Activity of Nicotinic Receptors Containing the Epsilon Subunit

NMJs of newborn animals contain both fetal (α_2_βγδ) and adult (α_2_βεδ) muscle nAChRs, and the switch from the fetal to the adult subtype occurs during the first two weeks in rodents [9,10,26]. Furthermore, Yumoto et al. [11] reported that, during the transitional period, γ- and ε-subunits are present simultaneously at individual endplates. Gu et al. [27] demonstrated that, in developing rats, end-plates between P9 and P16 contain both γ- and ε-subunits simultaneously. It is also known that the channel opening time differs markedly between fetal and adult receptors: 5.7 ms for fetal receptors vs. 1.6 ms for adult receptors [28].

On the basis of these findings, we hypothesized that blockade of CB1 receptors induces ACh release from an additional pool of synaptic vesicles located opposite the fields of fetal nAChRs, whose activation may increase the mean decay time constant. To test this hypothesis, we used Waglerin-1 (1 μM), which is a selective blocker of ε-subunit-containing muscle nAChRs [19]. Thus, after the exclusion of adult nAChRs with fast kinetics, the effect of AM-251 on QC was expected to be maintained; the decay time constant was not expected to be altered since the decay time constant does not depend on the number of open channels but reflects the kinetics of their closure.

**Figure 2:**
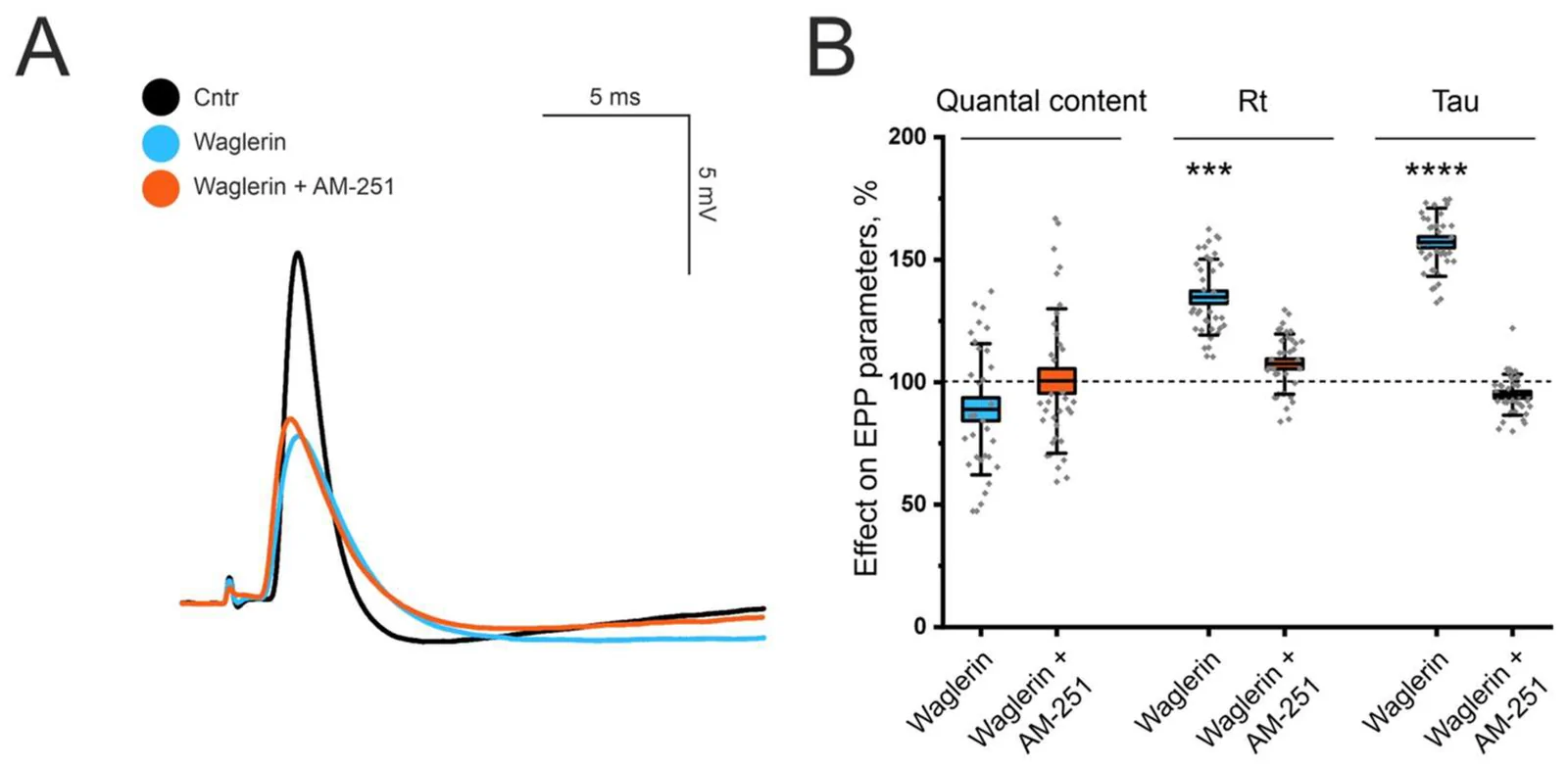
Action of the CB1 antagonist AM-251 on the parameters of evoked activity at the LAL NMJ in the presence of the ε-subunit-containing muscle nAChR blocker Waglerin-1 (1 μM) at P14. (**A**) Representative EPP recordings at NMJs in the control group (Cntr), Waglerin-1 alone and combined with AM-251-treated groups. (**B**) Effect of Waglerin-1 alone or in combination with AM-251 (5 μM) on EPP parameters (quantal content (QC), rise time (RT) and decay time constant (Tau)). *n* = 32–35 NMJs; N = 5 animals; *** *p* < 0.001; **** *p* < 0.0001 vs. control/blocker values (one-way ANOVA).

At P14, the application of Waglerin-1 did not alter the QC (88.96 ± 4.72%, *p* > 0.05; Figure 2A,B), although the mean rise time and decay time increased by 34.67 ± 2.55% (*p* < 0.001; Figure 2B) and 57.13 ± 2.25% (*p* < 0.001; Figure 2B), respectively. However, unexpectedly, the subsequent application of AM-251 (5 μM) did not affect the QC, decay time constant or rise time.

Thus, unexpectedly, both effects of AM-251 were eliminated in the presence of Waglerin-1. This observation suggests functional coupling between CB1 receptor signaling and muscle nAChRs such that blockade of the latter prevents the manifestation of CB1 receptor-mediated effects. The disappearance of the presynaptic effect on the QC following postsynaptic receptor blockade may indicate the existence of a retrograde signaling mechanism between the postsynaptic membrane and the motor nerve terminal. Such interactions could represent a form of synaptic homeostasis. Indeed, similar phenomena, whereby alterations in the number of active postsynaptic nAChRs lead to compensatory changes in presynaptic ACh release, have previously been described as synaptic homeostasis [29].

**Figure 3:**
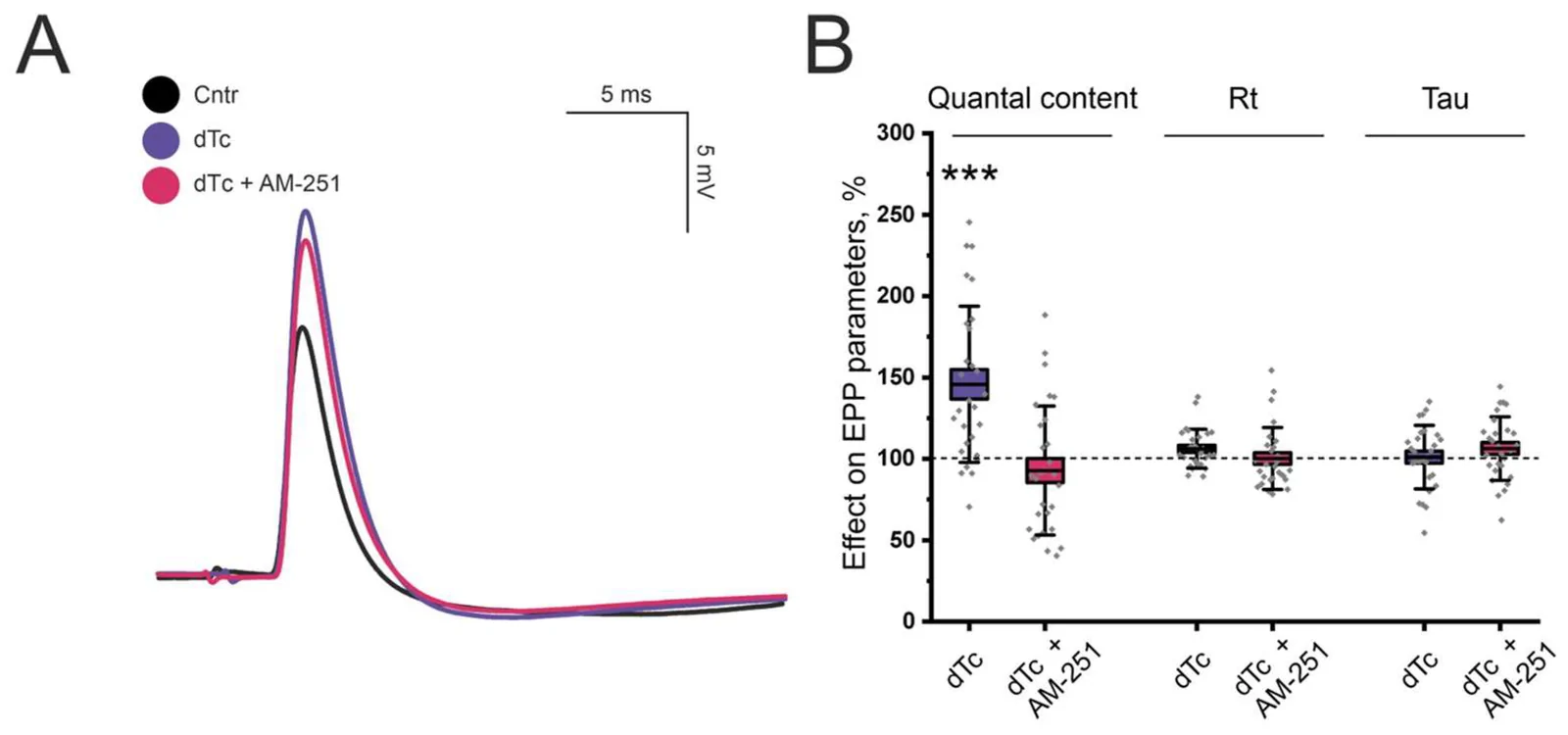
Action of the CB1 antagonist AM-251 on evoked activity at LAL NMJ in the presence of d-Tubocurarine (50 nM, dTc) at P14. (**A**) Representative EPP recordings at NMJs in the control group (Cntr), dTc alone or combined with AM-251 treatments. (**B**) Effect of dTc alone or combined with AM-251 (5 μM) on EPP parameters (quantal content (QC), rise time (RT) and decay time constant (Tau)). *n* = 33 NMJs; N = 5 animals; *** *p* < 0.001 vs. control/blocker values (one-way ANOVA).

To determine whether the observed phenomenon indeed represents a form of synaptic homeostasis, we next examined the effects of partial blockade of all muscle nAChR isoforms. To this end, we used dTc (50 nM), a selective antagonist of nAChRs [30]. The application of dTc *per se* significantly increased the QC by 45.73 ± 9.07% (*p* < 0.001; Figure 3B), as previously described [29]. However, neither the rise time nor the decay time was affected. Subsequent application of AM-251 (5 μM) produced no additional changes in the QC, rise time, or decay time constant. Thus, the effects of AM-251 depend on the density of active nAChR at the postsynaptic membrane.

**Figure 4:**
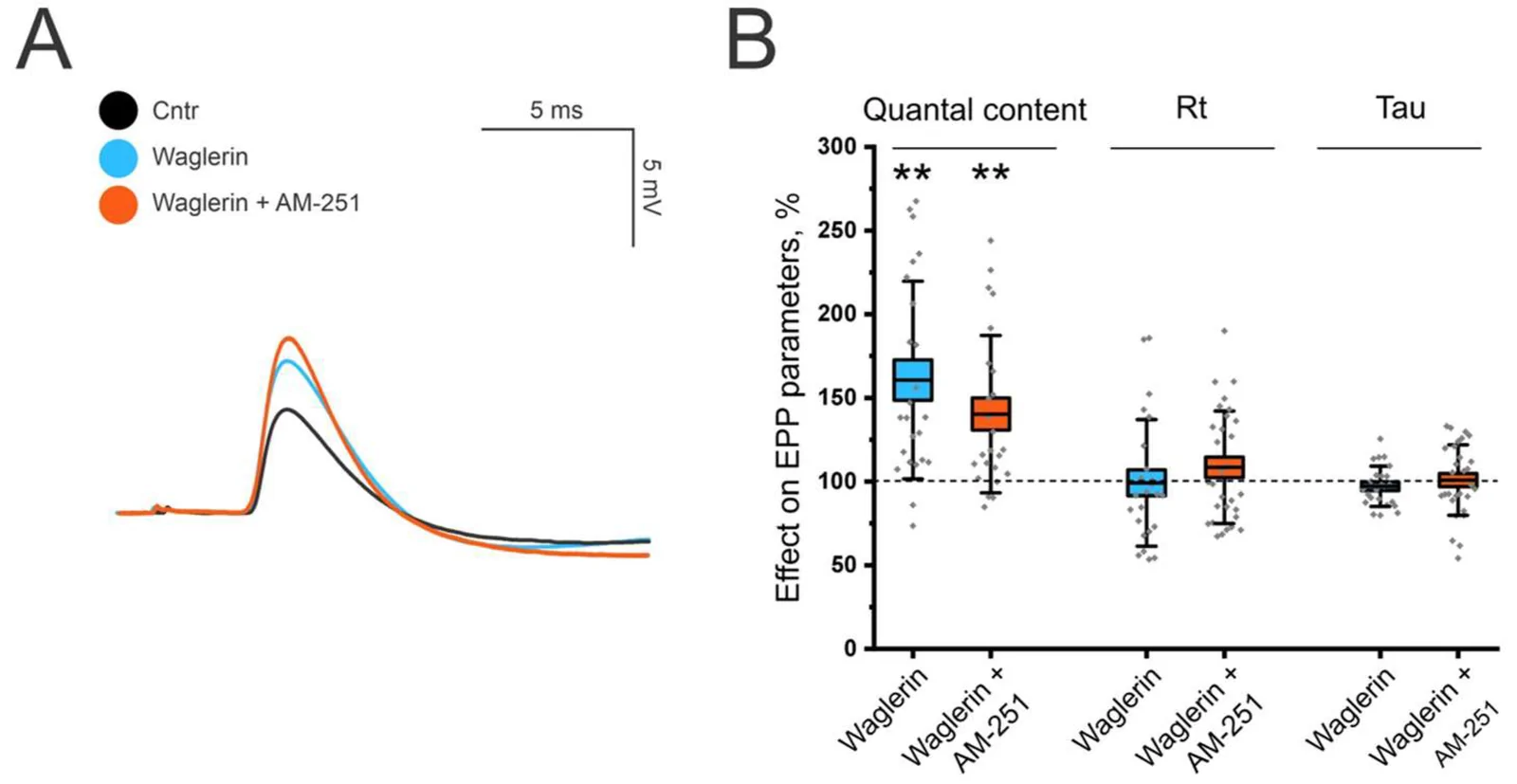
Action of the CB1 antagonist AM-251 on the parameters of evoked activity at the LAL NMJ in the presence of the ε-subunit-containing muscle nAChR blocker Waglerin-1 (1 μM) at P2. (**A**) Representative EPP recordings at NMJs in the control group (Cntr), Waglerin alone or combined with AM-251 treatment groups. (**B**) Effect of Waglerin alone or combined with AM-251 (5 μM) on EPP parameters (quantal content (QC), rise time (RT) and decay time constant (Tau)). *n* = 25 NMJs; N = 5 animals; ** *p* < 0.01 vs. control/blocker values (one-way ANOVA).

Next, we investigated how Waglerin-1 and the coapplication of Waglerin-1 with AM-251 affected the parameters of evoked ACh release in P2 aged mice. Waglerin-1 per se significantly increased the QC by 60.59 ± 12.05% (*p* < 0.05; Figure 4) and did not affect the rise time or decay time. When AM-251 was applied together with Waglerin-1, the QC increased by 40.3 ± 9.6% (*p* < 0.05; Figure 4), and neither the rise time nor the decay time were affected.

Thus, the data indicate that AM-251 continues to exert its modulatory effect on QC in the presence of Waglerin-1 but no longer affects the decay time constant. These findings suggest that a certain level of active adult muscle nAChR subtype may be required for the CB1 receptor-mediated modulation of the decay time constant.

### 3.3. Effects of CB1 Receptor Activation Depend on the Activity of Butyrylcholinesterase

2-AG is hydrolyzed by BChE in vitro, suggesting a possible role for BChE in eCB metabolism [31]. We decided to investigate whether such a non-canonical pathway for eCB degradation might also operate at NMJs ex vivo. To this end, we used bambuterol (100 nM) as a specific BChE inhibitor [32]. Notably, bambuterol is a prodrug of the selective long-acting b2-adrenoceptor agonist terbutaline, which is used for long-term treatment of bronchial asthma [33]. Bambuterol itself has no b2-adrenoceptor agonist effect, but it is metabolized by plasma BChE into bambuterol monocarbamate (inactive), which is further metabolized by the same enzyme into the pharmacologically active terbutaline [34]. During this time, BChE remains inhibited. Considering the extremely slow kinetics of the conversion of bambuterol to terbutaline [35], the effects of adrenergic receptor activation in our study can be neglected.

**Figure 5:**
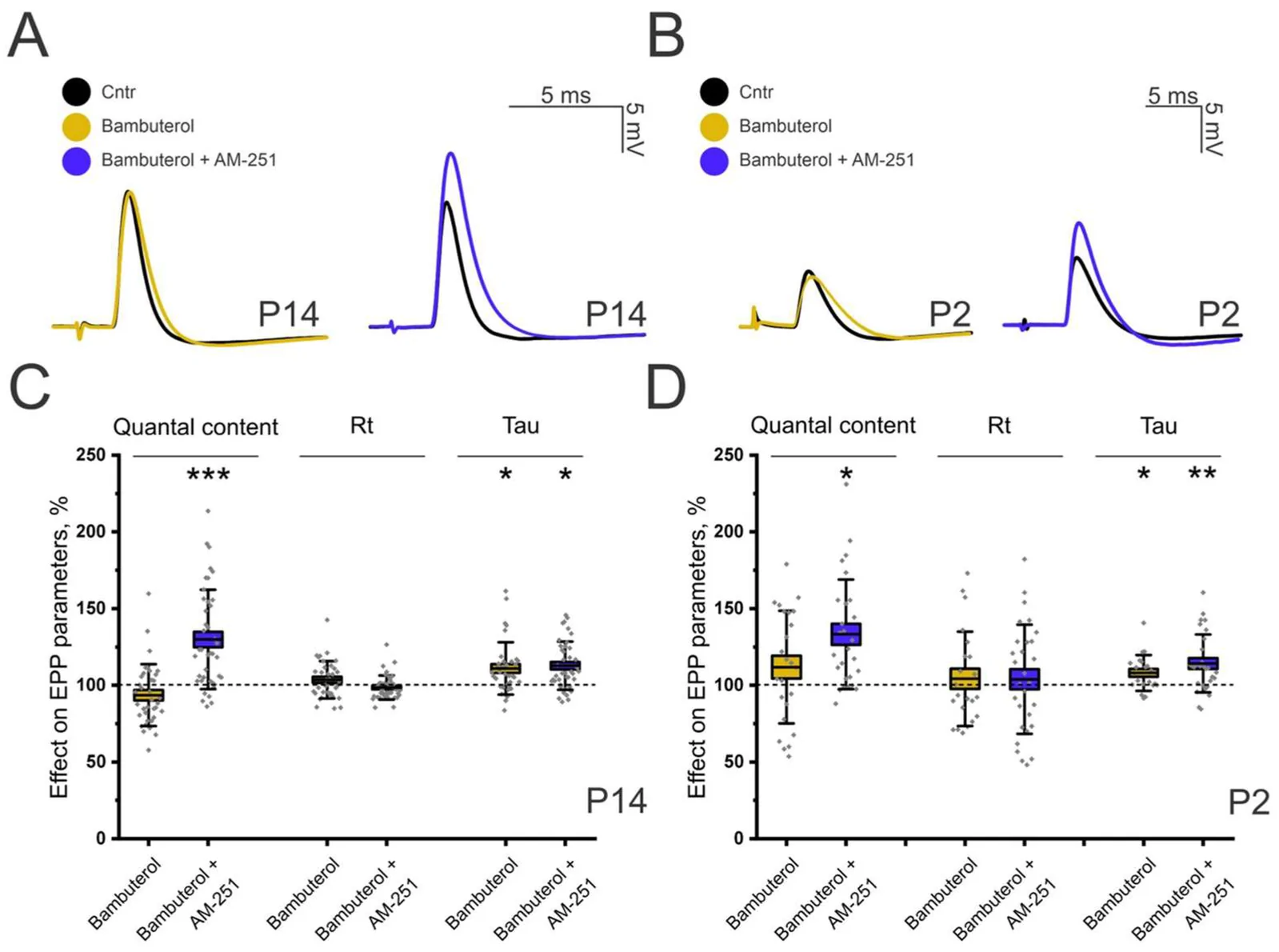
Action of the CB1 receptor antagonist AM-251 on EPP parameters at the LAL NMJ in the presence of the butyrylcholinesterase (BChE) blocker bambuterol (100 nM) at P14 and P2. (**A**,**B**) Representative EPP recordings at NMJs in the control group (Cntr) and following the application of bambuterol and subsequent AM-251 at P14 (left) and P2 (right). (**C**) Effect of AM-251 in the presence of bambuterol on EPP parameters at P14. (**D**) Effect of AM-251 in the presence of bambuterol on EPP parameters at P2. *n* = 24–41 NMJs; N = 5 animals; * *p* < 0.05, ** *p* < 0.01, *** *p* < 0.001, vs. control/blocker values (one-way ANOVA).

In young mice (P14), bambuterol had no effect on evoked ACh release (93.5 ± 3.4%, *p* = 0.9; Figure 5A,C); however, it increased the decay time constant by 10.94 ± 2.84% (*p* < 0.05; Figure 5A,C). A similar pattern was observed at P2, where the QC remained unchanged, but the decay time increased by 8.05 ± 2.54% (*p* < 0.05; Figure 5B,D). This slight increase in the EPP decay time in the presence of bambuterol may be explained by cholinesterase inhibition [36].

We then applied AM-251 (5 μM) to LAL muscles in which BChE had been pre-inhibited with bambuterol. In aged P14 mice, the effect of AM-251 was enhanced to 29.8 ± 5.1% (*p* < 0.001; Figure 5C), which was significantly greater than the effect of AM-251 *alone* (17.24 ± 4.9%, Figure 1C). However, the effect on decay time remained comparable to that of AM-251 alone and was 12.74 ± 2.45% (*p* < 0.05; Figure 5C).

In P2 animals, the application of AM-251 after bambuterol increased the QC and decay time by 33.24 ± 6.87% (*p* < 0.05; Figure 5D) and 14.2 ± 3.51% (*p* < 0.01; Figure 5D), respectively. These values did not differ significantly from those observed following the application of AM-251 alone (Figure 1B).

Thus, the increase in the effect of AM-251 on EPP QC following BChE inhibition at P14 suggests that BChE-sensitive processes can modulate eCB-dependent regulation of neurotransmitter release at NMJs.

**Figure 6:**
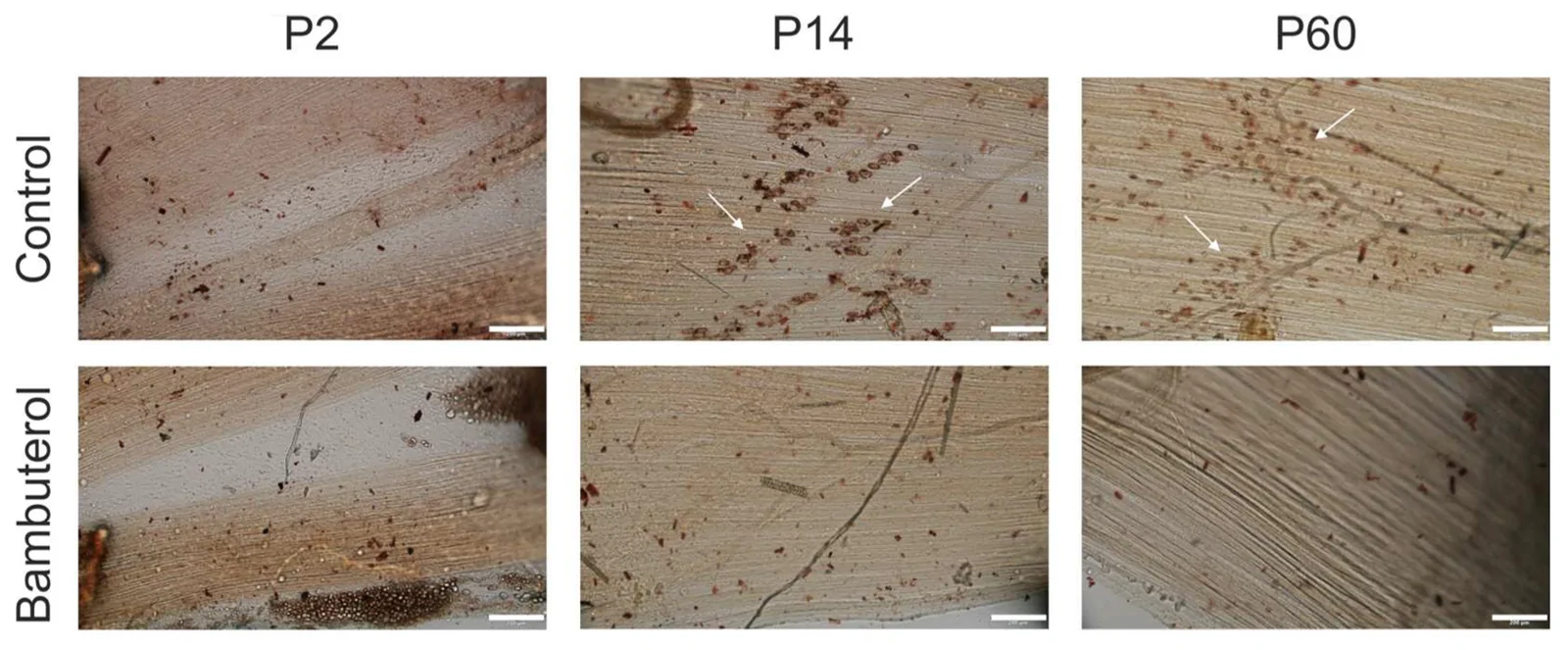
Histochemical detection of butyrylcholinesterase activity in the LAL muscles of newborn (P2), young (P14) and adult (P60) mice in Control and after the incubation with bambuterol (100 nM). The white arrows show an example of characteristic Hatchett’s brown precipitate (product of butyrylthiocholine hydrolysis). Scale bars, 200 µm. Representative images of muscles are obtained from a total of 5 muscles from 5 animals at each time point and in each group.

To verify the presence of extracellular BChE in LAL muscle, we performed histochemical detection of BChE activity by the method of Karnovsky [24]. To do this, the LAL muscles were incubated in a solution containing the specific BChE substrate butyrylthiocholine, which was hydrolyzed to thiocholine and acetic acid. The formed thiocholine binds to the ions of metals present in the solution, which leads to the precipitation of a characteristic color precipitate, known in histochemistry as Hatchett’s brown. The advantage of this method is that the precipitation occurs directly at the site of extracellular enzyme localization. Histochemical detection revealed no visible activity of BChE in the LAL muscle of aged P2 mice (Figure 6). However, characteristic histochemical staining was detected in the LAL of mice aged P14 and P60 (Figure 6). Notably, after preincubation of the muscles with bambuterol at a concentration of 100 nM, no visible activity of BChE was detected. Thus, bambuterol at this concentration significantly inhibited BChE activity.

### 3.4. CB1 Receptors Mediate the Modulation of the Force of Diaphragm Muscle Contraction

Changes in ACh release from motor nerve terminals may affect the physiological function of innervated muscle fibers. The hypothesis that eCB-induced regulation of ACh release can lead to a change in skeletal muscle contractile activity was tested using the tensiometry method.

**Figure 7:**
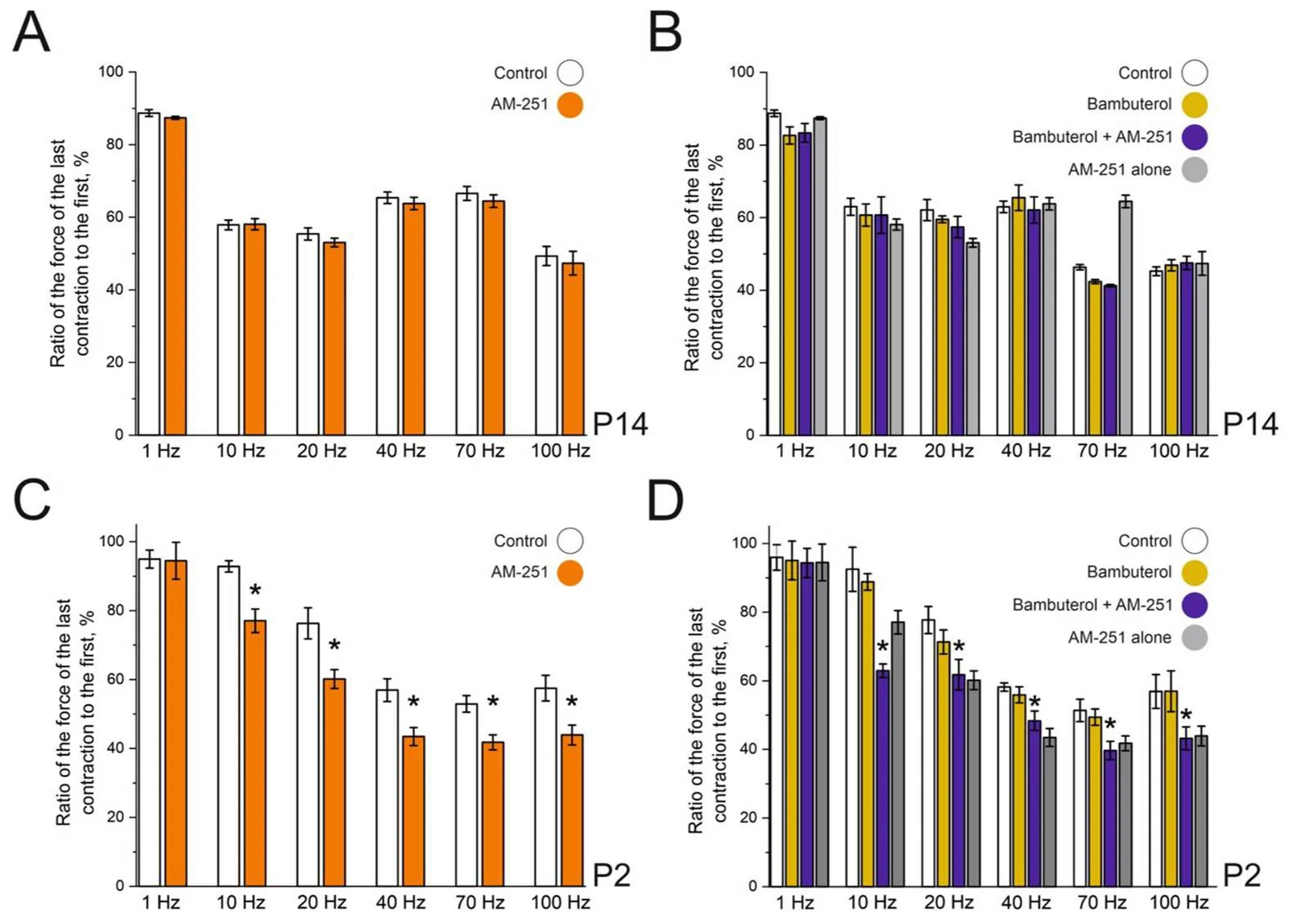
Effects of AM-251 (5 μM), bambuterol (100 nM) and AM-251 after bambuterol on the force of diaphragm muscle contraction at different frequencies of motor nerve stimulation (1–100 Hz) in P14 (**A**,**B**) and P2 (**C**,**D**) aged mice. *n* = 8 muscles; N = 8 animals; * *p* < 0.05.

In these experiments, the motor nerve was stimulated with a series of trains at frequencies of 1, 10, 20, 40, 70, and 100 Hz. The stimulation trains induced muscle fatigue, which was calculated as the ratio of the last contraction to the first contraction in the train (Figure 7).

In the diaphragm muscle at P14, the use of the CB1 receptor blocker AM-251 (Figure 7A) did not cause a significant change in the force of contraction compared with that in the control group. The addition of bambuterol or AM-251 after bambuterol treatment (Figure 7B) also did not significantly affect muscle fatigue.

At P2, the use of the CB1 receptor blocker AM-251 (5 μM) led to the development of pronounced frequency-dependent muscle fatigue. When stimulated at a frequency of 1 Hz, the level of muscle fatigue did not significantly change and remained at 94.49 ± 5.32% that of the control (*p* = 0.93). However, at a frequency of 10 Hz, the strength of the last contraction significantly decreased to 77.06 ± 3.42% (*p* = 0.00115). With increasing frequency of stimulation, the effect of AM-251 persisted: the strength of the last contraction decreased to 60.17 ± 2.73% at 20 Hz (*p* = 0.008), 43.49 ± 2.6% at 40 Hz (*p* = 0.0057), 41.78 ± 2.17% at 70 Hz (*p* = 0.0028) and 43.93 ± 2.87% at 100 Hz (*p* = 0.01).

The application of bambuterol (100 nM) did not affect the fatigue of the diaphragmatic muscle in P2-aged mice. However, the addition of AM-251 (5 μM) to bambuterol (100 nM) increased fatigue during stimulation at a frequency of 10 Hz, whereas AM-251 alone reduced the amplitude of the last response to 77.06 ± 3.42%, and the addition of bambuterol caused a decrease in contraction force to 62.94 ± 2.0% (*p* = 0.018 compared with AM-251 without bambuterol). At other stimulation frequencies, the effect of AM-251 in combination with bambuterol was the same as that of AM-251 alone.

Thus, the blockade of CB1 receptors increases the rate of diaphragm muscle fatigue in newborn mice, for example, due to the rapid consumption of the ready releasable pool of synaptic vesicles. At a frequency of 10 Hz, the effect of the CB1 receptor blocker increases in the presence of the BChE inhibitor. Thus, in contrast to LAL muscle, in the diaphragm muscle of P2 mice, BChE is involved in the CB1 receptor-mediated pathway.

## 4. Discussion

The main findings of this study are as follows: (i) endogenous regulation via CB1 receptors is present at mouse NMJs; (ii) functional muscle nAChRs are required for CB1 receptor-mediated effects; (iii) CB1 receptor blockade leads to an increase in the decay time, and the implementation of this phenomenon depends on the adult form of muscle nAChR; and (iv) the effect of eCB can be enhanced by BChE blockade.

Tonic eCB regulation is now recognized as a common feature of synaptic transmission throughout the nervous system. In the CNS, the constitutive activation of presynaptic CB1 receptors by endogenously produced cannabinoids provides negative feedback that limits neurotransmitter release and contributes to both short- and long-term synaptic plasticity [4,37,38,39]. During development, endocannabinoid signaling additionally regulates axonal growth, synapse formation and circuit refinement [1,12,40]. Similar inhibitory CB1 receptor-dependent mechanisms have been described in several peripheral neuroeffector junctions. In sympathetic nerves, the activation of presynaptic CB1 receptors suppresses noradrenaline release [41], whereas in the enteric nervous system, CB1 receptors expressed by cholinergic myenteric neurons inhibit cholinergic neurotransmission and gastrointestinal motility [42,43]. In contrast, evidence for eCB signaling at mammalian NMJs has remained extremely limited. Previous studies have focused primarily on the actions of exogenously applied cannabinoids, whereas physiological tonic regulation by eCB has been demonstrated only in the lizard NMJ [8]. Our findings therefore extend this principle to the developing mammalian NMJ, as endogenous CB1 signaling tonically restrains ACh release during early postnatal development.

Homeostatic synaptic plasticity is a compensatory mechanism that maintains stable synaptic transmission in response to perturbations such as partial blockade of postsynaptic ACh receptors. This phenomenon was first described approximately four decades ago at the vertebrate NMJ [44,45,46,47,48,49]. It has since been extensively studied at the NMJ in *Drosophila melanogaster*, where postsynaptic receptor blockade triggers a compensatory increase in the QC accompanied by increased presynaptic Ca^2+^ influx [50,51,52]. Emerging evidence further suggests that eCB signaling contributes to the homeostatic regulation of synaptic transmission. In hippocampal neurons, prolonged tetrodotoxin treatment induces homeostatic strengthening of excitatory synapses through CB1 receptor-dependent modulation of eCB signaling [53]. Similarly, increased AEA signaling and enhanced CB1 receptor expression have been shown to stabilize neuronal network activity during chronic hyperexcitability [54], whereas reduced eCB tone represents an independent homeostatic mechanism regulating GABA release [55]. Together, these findings suggest that eCB signaling may constitute an additional component of the molecular machinery underlying homeostatic regulation of developing NMJ.

Modulation of nAChR channel kinetics is a complicated process. For example, nAChR activity in parasympathetic neurons can be directly regulated by G proteins through interactions with the intracellular domains of these receptors [56]. In addition, hormones such as hydrocortisone have been shown to modulate nAChR channel kinetics [57]. Moreover, co-neurotransmitters such as ADP and calcitonin gene-related peptide can modulate the activity of embryonic nAChRs through phosphorylation of specific receptor sites [58]. Taken together, these findings suggest that the modulation of nAChR kinetics represents a common mechanism for regulating synaptic transmission. Therefore, the modulation of EPP decay time observed in the present study may represent an additional mechanism regulating synaptic transmission during NMJ development. Such modulation could alter the temporal profile of nAChR activation and, consequently, the duration of postsynaptic signaling during the critical period of NMJ maturation.

eCBs (2-AG and AEA) are primarily degraded by monoacylglycerol lipase and fatty acid amide hydrolase, respectively, representing the canonical pathways of eCB metabolism [59]. However, several non-canonical metabolic pathways involving enzymes such as membrane-associated serine hydrolases (ABHD6 and ABHD12), cytochrome P450, lipoxygenases, N-acylethanolamine acid amidase, and cyclooxygenase-2 have been described [60,61,62]. To date, there is no evidence that eCB undergo extracellular hydrolysis under physiological conditions. Nevertheless, an in vitro study demonstrated that 2-AG can be hydrolyzed by BChE, suggesting that this enzyme may contribute to eCB metabolism and the extracellular regulation of eCB signaling [31]. Our findings demonstrate that pharmacological inhibition of BChE potentiates the effects of AM-251, suggesting that BChE may regulate tonic eCB signaling at the developing NMJ. These observations are consistent with two interrelated possibilities: (i) the existence of an extracellular pathway for endocannabinoid degradation and/or (ii) the presence of a BChE-sensitive mechanism involved in CB1 receptor-dependent regulation of synaptic transmission.

This study has several limitations that should be acknowledged. Bambuterol may exhibit off-target effects at the concentration employed. The gradual conversion of Bambuterol to the β2-adrenoceptor agonist terbutaline [33,34] could theoretically influence muscle fatigue profiles. Furthermore, the ex vivo isolated nerve–muscle preparation, while enabling precise electrophysiological and tensometric recordings, lacks information concerning systemic neurohumoral regulation and central motor drive. Unavoidable alterations in baseline synaptic transmission and endocannabinoid tone caused by nerve preparation procedures may also affect the interpretation of the results. Future studies utilizing genetic knockout models and in vivo approaches are warranted to validate and extend these findings.

## 5. Conclusions

In summary, this study revealed that CB1 receptors mediate a transient, developmentally restricted modulation of ACh release and probably nAChR kinetics at the NMJ, which disappears in adulthood. We demonstrate that eCB signaling is functionally coupled to postsynaptic adult-type nAChR activity, suggesting a novel retrograde homeostatic mechanism. Furthermore, we identified BChE as an active player in the process of eCB-mediated regulation of QC, most likely via eCB degradation. Collectively, our findings reveal an interplay between the eCB system and postsynaptic nAChR. This interplay seems essential for maintaining optimal neuromuscular synaptic transmission and muscle performance during postnatal development.

## Data Availability

The original contributions presented in this study are included in the article. Further inquiries can be directed to the corresponding authors.

## Abbreviations

The following abbreviations are used in this manuscript:

ACh	Acetylcholine
ADP	Adenosine diphosphate
AEA	Anandamide
2-AG	2-arachidonoylglycerol
BChE	Butyrylcholinesterase
Cntr	Control
dTc	d-tubocurarine
eCB	Endocannabinoid
EPP	End-plate potential
LAL	Levator auris longus
NMJ	Neuromuscular junction
nAChR	Nicotinic acetylcholine receptor
P	Postnatal day
QC	Quantal content
mEPP	Miniature end-plate potential
SEM	Standard error

## References

[1] Fride E. (2008). Multiple Roles for the Endocannabinoid System During the Earliest Stages of Life: Pre- and Postnatal Development. J. Neuroendocrinol..

[2] Bukiya A.N. (2019). Physiology of the Endocannabinoid System During Development. Adv. Exp. Med. Biol..

[3] Correa F., Wolfson M.L., Valchi P., Aisemberg J., Franchi A.M. (2016). Endocannabinoid System and Pregnancy. Reproduction.

[4] Castillo P.E., Younts T.J., Chávez A.E., Hashimotodani Y. (2012). Endocannabinoid Signaling and Synaptic Function. Neuron.

[5] Van der Kloot W. (1994). Anandamide, a Naturally-Occurring Agonist of the Cannabinoid Receptor, Blocks Adenylate Cyclase at the Frog Neuromuscular Junction. Brain Res..

[6] Silveira P.E., Silveira N.A., de Cássia Morini V., Kushmerick C., Naves L.A. (2010). Opposing Effects of Cannabinoids and Vanilloids on Evoked Quantal Release at the Frog Neuromuscular Junction. Neurosci. Lett..

[7] Tarasova E.O., Khotkina N.A., Bogacheva P.O., Chernyshev K.A., Gaydukov A.E., Balezina O.P. (2022). Noncanonical Potentiation of Evoked Quantal Release of Acetylcholine by Cannabinoids Anandamide and 2-Arachidonoylglycerol in Mouse Motor Synapses. Biochem. Mosc. Suppl. Ser. A.

[8] Newman Z., Malik P., Wu T., Ochoa C., Watsa N., Lindgren C. (2007). Endocannabinoids Mediate Muscarine-induced Synaptic Depression at the Vertebrate Neuromuscular Junction. Eur. J. Neurosci..

[9] Hall Z., Gorin P., Silberstein L., Bennett C. (1985). A Postnatal Change in the Immunological Properties of the Acetylcholine Receptor at Rat Muscle Endplates. J. Neurosci..

[10] Missias A.C., Chu G.C., Klocke B.J., Sanes J.R., Merlie J.P. (1996). Maturation of the Acetylcholine Receptor in Skeletal Muscle: Regulation of the AChR γ-to-ϵ Switch. Dev. Biol..

[11] Yumoto N., Wakatsuki S., Sehara-Fujisawa A. (2005). The Acetylcholine Receptor γ-to-ε Switch Occurs in Individual Endplates. Biochem. Biophys. Res. Commun..

[12] Watson S., Chambers D., Hobbs C., Doherty P., Graham A. (2008). The Endocannabinoid Receptor, CB1, Is Required for Normal Axonal Growth and Fasciculation. Mol. Cell. Neurosci..

[13] An D., Peigneur S., Hendrickx L.A., Tytgat J. (2020). Targeting Cannabinoid Receptors: Current Status and Prospects of Natural Products. Int. J. Mol. Sci..

[14] Lan R., Liu Q., Fan P., Lin S., Fernando S.R., McCallion D., Pertwee R., Makriyannis A. (1999). Structure−Activity Relationships of Pyrazole Derivatives as Cannabinoid Receptor Antagonists. J. Med. Chem..

[15] Baur R., Gertsch J., Sigel E. (2012). The Cannabinoid CB1 Receptor Antagonists Rimonabant (SR141716) and AM251 Directly Potentiate GABA A Receptors. Br. J. Pharmacol..

[16] Tunek A., Svensson L.A. (1988). Bambuterol, a Carbamate Ester Prodrug of Terbutaline, as Inhibitor of Cholinesterases in Human Blood. Drug Metab. Dispos..

[17] Willcockson I.U., Hong A., Whisenant R.P., Edwards J.B., Wang H., Sarkar H.K., Pedersen S.E. (2002). Orientation of D-Tubocurarine in the Muscle Nicotinic Acetylcholine Receptor-Binding Site. J. Biol. Chem..

[18] Le Dain A.C., Madsen B.W., Edeson R.O. (1991). Kinetics of (+)-tubocurarine Blockade at the Neuromuscular Junction. Br. J. Pharmacol..

[19] McArdle J.J., Lentz T.L., Witzemann V., Schwarz H., Weinstein S.A., Schmidt J.J. (1999). Waglerin-1 Selectively Blocks the Epsilon Form of the Muscle Nicotinic Acetylcholine Receptor. J. Pharmacol. Exp. Ther..

[20] Nevsky E., Sibgatullina G., Samigullin D., Malomouzh A., Parpura V., Petrov K. (2025). Age-Dependent Regulation of Acetylcholine Release at the Neuromuscular Junction Mediated by GABA. Cells.

[21] Bazzy A.R. (1994). Developmental Changes in Rat Diaphragm Endplate Response to Repetitive Stimulation. Dev. Brain Res..

[22] Bogacheva P.O., Potapova D.A., Gaydukov A.E. (2025). Sortilin and L-Type Calcium Channels May Be Involved in the Unusual Mechanism of ProBDNF Signaling in Regenerating Mouse Neuromuscular Junctions. Neurochem. Res..

[23] McLachlan E.M., Martin A.R. (1981). Non-linear Summation of End-plate Potentials in the Frog and Mouse. J. Physiol..

[24] Kugler P. (1987). Improvement of the Method of Karnovsky and Roots for the Histochemical Demonstration of Acetylcholinesterase. Histochemistry.

[25] Petrov K.A., Kharlamova A.D., Lenina O.A., Nurtdinov A.R., Sitdykova M.E., Ilyin V.I., Zueva I.V., Nikolsky E.E. (2018). Specific Inhibition of Acetylcholinesterase as an Approach to Decrease Muscarinic Side Effects during Myasthenia Gravis Treatment. Sci. Rep..

[26] Sakmann B., Brenner H.R. (1978). Change in Synaptic Channel Gating during Neuromuscular Development. Nature.

[27] Gu Y., Hall Z.W. (1988). Immunological Evidence for a Change in Subunits of the Acetylcholine Receptor in Developing and Denervated Rat Muscle. Neuron.

[28] Kopta C., Steinbach J. (1994). Comparison of Mammalian Adult and Fetal Nicotinic Acetylcholine Receptors Stably Expressed in Fibroblasts. J. Neurosci..

[29] Wang X., McIntosh J.M., Rich M.M. (2018). Muscle Nicotinic Acetylcholine Receptors May Mediate Trans-Synaptic Signaling at the Mouse Neuromuscular Junction. J. Neurosci..

[30] Griffith H.R., Johnson G.E. (1942). The use of curare in general anesthesia. Anesthesiology.

[31] Barricklow J., Blatnik M. (2013). 2-Arachidonoylglycerol Is a Substrate for Butyrylcholinesterase: A Potential Mechanism for Extracellular Endocannabinoid Regulation. Arch. Biochem. Biophys..

[32] Minic J., Chatonnet A., Krejci E., Molgó J. (2003). Butyrylcholinesterase and Acetylcholinesterase Activity and Quantal Transmitter Release at Normal and Acetylcholinesterase Knockout Mouse Neuromuscular Junctions. Br. J. Pharmacol..

[33] Sitar D.S. (1996). Clinical Pharmacokinetics of Bambuterol. Clin. Pharmacokinet..

[34] Pistolozzi M., Du H., Wei H., Tan W. (2015). Stereoselective Inhibition of Human Butyrylcholinesterase by the Enantiomers of Bambuterol and Their Intermediates. Drug Metab. Dispos..

[35] Tunek A., Levin E., Svensson L.-Å. (1988). Hydrolysis of 3H-Bambuterol, a Carbamate Prodrug of Terbutaline, in Blood from Humans and Laboratory Animals in Vitro. Biochem. Pharmacol..

[36] Katz B., Miledi R. (1973). The Binding of Acetylcholine to Receptors and Its Removal from the Synaptic Cleft. J. Physiol..

[37] Kreitzer A.C., Regehr W.G. (2001). Retrograde Inhibition of Presynaptic Calcium Influx by Endogenous Cannabinoids at Excitatory Synapses onto Purkinje Cells. Neuron.

[38] Ohno-Shosaku T., Maejima T., Kano M. (2001). Endogenous cannabinoids mediate retrograde signals from depolarized postsynaptic neurons to presynaptic terminals. Neuron.

[39] Chevaleyre V., Takahashi K.A., Castillo P.E. (2006). Endocannabinoid-mediated synaptic plasticity in the CNS. Annu. Rev. Neurosci..

[40] Harkany T., Keimpema E., Barabás K., Mulder J. (2008). Endocannabinoid Functions Controlling Neuronal Specification during Brain Development. Mol. Cell. Endocrinol..

[41] Ishac E.J.N., Jiang L., Lake K.D., Varga K., Abood M.E., Kunos G. (1996). Inhibition of Exocytotic Noradrenaline Release by Presynaptic Cannabinoid CB1 Receptors on Peripheral Sympathetic Nerves. Br. J. Pharmacol..

[42] Izzo A.A., Coutts A.A. (2005). Cannabinoids and the Digestive Tract. Handbook of Experimental Pharmacology.

[43] Coutts A.A., Irving A.J., Mackie K., Pertwee R.G., Anavi-Goffer S. (2002). Localisation of Cannabinoid CB1 Receptor Immunoreactivity in the Guinea Pig and Rat Myenteric Plexus. J. Comp. Neurol..

[44] Katz B., Miledi R. (1978). A Re-Examination of Curare Action at the Motor Endplate. Proc. R. Soc. Lond. B Biol. Sci..

[45] Cull-Candy S.G., Miledi R., Trautmann A., Uchitel O.D. (1980). On the Release of Transmitter at Normal, Myasthenia Gravis and Myasthenic Syndrome Affected Human End-plates. J. Physiol..

[46] Wilson D.F. (1982). Influence of Presynaptic Receptors on Neuromuscular Transmission in Rat. Am. J. Physiol.-Cell Physiol..

[47] Plomp J.J., van Kempen G.T., Molenaar P.C. (1992). Adaptation of Quantal Content to Decreased Postsynaptic Sensitivity at Single Endplates in Alpha-bungarotoxin-treated Rats. J. Physiol..

[48] Tian L., Prior C., Dempster J., Marshall I.G. (1994). Nicotinic Antagonist-produced Frequency-dependent Changes in Acetylcholine Release from Rat Motor Nerve Terminals. J. Physiol..

[49] Wang X., Wang Q., Engisch K.L., Rich M.M. (2010). Activity-Dependent Regulation of the Binomial Parameters p and n at the Mouse Neuromuscular Junction In Vivo. J. Neurophysiol..

[50] Frank C.A., Kennedy M.J., Goold C.P., Marek K.W., Davis G.W. (2006). Mechanisms Underlying the Rapid Induction and Sustained Expression of Synaptic Homeostasis. Neuron.

[51] Frank C.A., Pielage J., Davis G.W. (2009). A Presynaptic Homeostatic Signaling System Composed of the Eph Receptor, Ephexin, Cdc42, and CaV2.1 Calcium Channels. Neuron.

[52] Müller M., Davis G.W. (2012). Transsynaptic Control of Presynaptic Ca2+ Influx Achieves Homeostatic Potentiation of Neurotransmitter Release. Curr. Biol..

[53] Song Y., Zhang J., Chen C. (2015). Fine-Tuning of Synaptic Upscaling at Excitatory Synapses by Endocannabinoid Signaling Is Mediated via the CB1 Receptor. Sci. Rep..

[54] Ye M., Monroe S.K., Gay S.M., Armstrong M.L., Youngstrom D.E., Urbina F.L., Gupton S.L., Reisdorph N., Diering G.H. (2022). Coordinated Regulation of CB1 Cannabinoid Receptors and Anandamide Metabolism Stabilizes Network Activity during Homeostatic Downscaling. eNeuro.

[55] Kim J., Alger B.E. (2010). Reduction in Endocannabinoid Tone Is a Homeostatic Mechanism for Specific Inhibitory Synapses. Nat. Neurosci..

[56] Fischer H., Liu D.-M., Lee A., Harries J.C., Adams D.J. (2005). Selective Modulation of Neuronal Nicotinic Acetylcholine Receptor Channel Subunits by Go-Protein Subunits. J. Neurosci..

[57] Nurowska E., Ruzzier F. (2002). Modulation of Acetylcholine Receptor Channel Kinetics by Hydrocortisone. Biochim. Biophys. Acta (BBA) -Biomembr..

[58] Liou J., Fu W. (1995). Additive Effect of ADP and CGRP in Modulation of the Acetylcholine Receptor Channel in Xenopus Embryonic Myocytes. Br. J. Pharmacol..

[59] Ueda N., Tsuboi K., Uyama T. (2013). Metabolism of Endocannabinoids and Related N-acylethanolamines: Canonical and Alternative Pathways. FEBS J..

[60] Urquhart P., Nicolaou A., Woodward D.F. (2015). Endocannabinoids and Their Oxygenation by Cyclo-Oxygenases, Lipoxygenases and Other Oxygenases. Biochim. Biophys. Acta (BBA) -Mol. Cell Biol. Lipids.

[61] Lu H.-C., Mackie K. (2016). An Introduction to the Endogenous Cannabinoid System. Biol. Psychiatry.

[62] Cao J.K., Kaplan J., Stella N. (2019). ABHD6: Its Place in Endocannabinoid Signaling and Beyond. Trends Pharmacol. Sci..

